# Suppression of pancreatic cancer proliferation through TXNIP-mediated inhibition of the MAPK signaling pathway: TXNIP inhibits pancreatic cancer via the MAPK pathway

**DOI:** 10.3724/abbs.2023286

**Published:** 2024-01-16

**Authors:** Qinglin Fei, Kaizhou Jin, Saimeng Shi, Tianjiao Li, Duancheng Guo, Mengxiong Lin, Xianjun Yu, Weiding Wu, Longyun Ye

**Affiliations:** 1 Department of Pancreatic Surgery Fudan University Shanghai Cancer Center Shanghai 200032 China; 2 Department of Oncology Shanghai Medical College Fudan University Shanghai 200032 China; 3 Shanghai Pancreatic Cancer Institute Shanghai 200032 China; 4 Pancreatic Cancer Institute Fudan University Shanghai 200032 China

**Keywords:** TXNIP, pancreatic cancer, MAPK signaling pathway

## Abstract

Thioredoxin-interacting protein (TXNIP) is a crucial thioredoxin-binding protein that is recognized as a tumor suppressor in diverse malignancies, such as breast cancer, lung cancer, hepatocellular carcinoma, and thyroid cancer. However, the specific role and molecular mechanisms of TXNIP in the pathogenesis and progression of pancreatic cancer cells have not been determined. In this study, we investigate the relationship between TXNIP expression and overall survival prognosis in pancreatic cancer patients. Mechanistic studies are conducted to reveal the role of TXNIP in pancreatic cancer cell proliferation, migration, and regulation during malignancy. Our findings indicate that patients with high TXNIP expression have a more favorable prognosis. *In vitro* experiments with pancreatic cell lines show that overexpression of TXNIP suppresses the proliferation and migration of pancreatic cancer cells. Furthermore, we find that TXNIP inhibits the activation of the MAPK signaling pathway, thereby decreasing the malignant potential of pancreatic cancer. In conclusion, our study reveals TXNIP as a promising new predictive marker and therapeutic target for pancreatic cancer.

## Introduction

Pancreatic cancer is one of the most lethal malignancies worldwide and is characterized by aggressive tumor progression, late-stage diagnosis, and resistance to conventional therapies [ 1– 3]. The complex interplay of genetic and epigenetic alterations contributes to the dysregulation of critical pathways involved in pancreatic carcinogenesis [ 4 – 6], emphasizing the need for novel molecular targets and therapeutic strategies [ 7, 8]. 

Thioredoxin-interacting protein (TXNIP) is a member of the alpha arrestin protein family and serves as a key interlocutor within the thioredoxin (TXN) antioxidant system [9]. TXNIP plays a pivotal role in regulating the levels of reactive oxygen species (ROS) and promoting inflammatory responses, and is an important factor in intracellular oxidative stress responses [ 10 – 12]. Previous evidence has shown that TXNIP expression is characteristically downregulated in a spectrum of cancers, including lung cancer, hepatic carcinoma, and gastric malignancies [ 13– 15]. The tumor-suppressive attributes of TXNIP are manifested in several ways. In breast cancer, TXNIP can suppress cell proliferation via metabolic reprogramming and attenuate cell invasion and migration through the TXNIP-HIF1α-TWIST signaling pathway [16]. In bladder cancer, TXNIP can promote apoptosis by inhibiting Erk protein activation [16]. These observations suggest that suppression of TXNIP expression may facilitate the malignant transformation of tumor cells, whereas enhancement of TXNIP expression could influence the metabolic state of tumor cells, thereby inhibiting their proliferative, invasive, and other malignant potentials. However, the specific role and potential mechanisms of TXNIP in pancreatic cancer remain largely unclarified. 

In this study, we investigated the role of TXNIP in pancreatic cancer. Our results highlighted the key role of TXNIP in suppressing tumor cell proliferation and mitigating metastasis. We also revealed that these anticarcinogenic properties stem from modulation of the MAPK signaling pathway, particularly through the dephosphorylation of Erk1/2. Overall, this study reveals new prognostic markers and potential therapeutic targets for pancreatic cancer.

## Materials and Methods

### Patients and tissue samples

Human pancreatic cancer tissues were collected from patients who underwent surgery at Fudan University Shanghai Cancer Center (Shanghai, China) from November 2011 to May 2014. Pathologically confirmed pancreatic ductal adenocarcinoma (PDAC) was confirmed in all patients. Patients who received neoadjuvant treatment or had inflammatory disease or active infection were excluded. A total of 218 patients diagnosed with PDAC were enrolled. The evaluated data included sex, age, tumor location, tumor diameter, CA19-9 concentration, TNM stage, and tumor grade. Tumor staging was performed in accordance with the American Joint Committee on Cancer TNM classification (8th edition). Overall survival (OS) was defined as the period from the date of surgery to the date of death. The study was approved by the ethics committee at FUSCC, and all patients provided written informed consent in accordance with the principles of the Declaration of Helsinki.

### Tissue microarray and immunohistochemical staining

TMAs were generated by Wuhan Servicebio Technology (Wuhan, China). The precise localization of PDAC lesions within specimens was assessed by hematoxylin and eosin (H&E) staining, and representative cylinders were punched away from necrotic and hemorrhagic materials. The procedure used for immunohistochemical (IHC) staining was described in our previous research [17]. The expression level of TXNIP was assessed by employing the histopathological scoring schema as defined by Jennifer *et al*. [18], with a benchmark score of ≥2 indicating positive expression. An exhaustive examination of all specimens was independently conducted by two experienced pathologists who were blinded to the clinical data. 

### Cell culture

The human pancreatic cancer cell lines PANC-1, SW1990, BxPC-3 and Capan-1 were purchased from the Cell Bank, Type Culture Collection, Chinese Academy of Sciences (Shanghai, China). The cell lines were meticulously maintained in accordance with the established protocols as stipulated by the provider. All cell lines were authenticated by genotyping performed by the Cell Bank of the Chinese Academy of Sciences and were tested to rule out mycoplasma contamination.

### Plasmid

The overexpression plasmid was constructed by incorporating cDNA encoding the *TXNIP* gene into the CMV-MCS-EF1α-Puro vector (Addgene plasmid 72265; Addgene, Watertown, USA). To downregulate TXNIP expression, a 21 bp target against TXNIP was incorporated into the pLKO.1. The TRC cloning vector (Addgene plasmid 10878; Addgene) and scramble shRNA (Addgene plasmid 1864; Addgene) were used. The sequences are shown in Table 1. Then, lentiviruses carrying the vectors mentioned above were constructed and used to infect PANC-1, SW1990 and BXPC-3 cells separately to generate stably transfected cell lines.

**Table:** **Table 1** shRNA sequences used in this study

Target	Sequence (5′→3′)
TXNIP_shRNA1_forward	CCGGCCCTGCTATATGGATGTCATTCTCGAGAATGACATCCATATAGCAGGGTTTTTG
TXNIP_shRNA1_reverse	AATTCAAAAACCCTGCTATATGGATGTCATTCTCGAGAATGACATCCATATAGCAGGG
TXNIP_shRNA2_forward	CCGGCCGACTTATACTGAGGTGGATCTCGAGATCCACCTCAGTATAAGTCGGTTTTTG
TXNIP_shRNA2_reverse	AATTCAAAAACCGACTTATACTGAGGTGGATCTCGAGATCCACCTCAGTATAAGTCGG

### Chemicals

The ferroptosis inducers RSL3, erastin [an Erk1/2 inhibitor (PD98059)], and gemcitabine were purchased from MedChemExpress (Monmouth Junction, USA).

### Cell viability and cell apoptosis assay

Cells (1×10 ^4^) were placed in 96-well plates and then subject to specific chemical treatments. After a 24‒72 h incubation period, cell viability was evaluated using a Cell Counting Kit-8 (Selleck, Houston, USA), and detection was facilitated with the Synergy H4 system (BioTek, Winooski, USA). An Annexin V 633 Apoptosis Detection kit (Dojindo, Tokyo, Japan) and a FACS system (Beckman Coulter, Pasadena, USA) were used to assess cell apoptosis. 

### EdU assay

The EdU (5-ethynyl-2′-deoxyuridine) assay was performed using EdU Cell Proliferation kit (BeyoClick, Shanghai, China) with Alexa Fluor 488 according to the manufacturer’s instructions. The PANC-1,SW1990, BxPC-3 and Capan-1 cells were incubated for 2 h with 10 μm EdU medium and then fixed with 4% paraformaldehyde. The click reaction solution was added and incubated for 30 min. EdU-positive cells were visualized and imaged using the fluorescence microscope. EdU-positive cells were counted in 10 different microscopic fields (magnification, 100×) in a random manner and analyzed using ImageJ software (NIH, Bethesda, USA).

### Colony formation assay

Cells (1×10 ^3^) were plated in 6 well plates and cultured with 5% CO _2_ at 37 °C for 2 weeks. The colonies were fixed with 4% methanol for 5 min and stained with 0.5% crystal violet for 10 min at room temperature for photographing and counting the number of colonies. 

### Transwell migration assay

A 24-well Transwell chamber with an 8-μm-pore PET membrane (BD Biosciences, Franklin Lakes, USA) was used to conduct migration assays. The lower chamber was filled with 400 μL media containing 10% FBS. Subsequently, approximately 10 ^5^ cells were seeded in 200 μL medium without serum in the top chamber. The cells migrated at 37°C with 5% CO _2_ over 24 h. After removal of the nonmigrated cells, the remaining cells were washed, fixed, and stained with crystal violet. The number of migrating cells was counted in six fields randomly selected at magnification ×100. 

### RNA extraction and real-time quantitative PCR

Total RNA was extracted utilizing Trizol reagent (Invitrogen, Carlsbad, USA), after which the isolated RNA was reverse transcribed into cDNA with the Vazyme HiScript III RT SuperMix Reagent kit (Vazyme, Nanjing, China). The expression levels of the target gene and the housekeeping gene *ACTB* were quantified using RT-qPCR. Each experiment was conducted in triplicate to ensure data reliability. The specific sequences of primers used are shown in Table 2.

**Table:** **Table 2** Sequences of primers for RT-qPCR used in this study

Name	Sequence (5′→3′)
*TXNIP*_ forward	AAGCAGCAGAACATCCAGC
*TXNIP*_ reverse	TGCAGGGATCCACCTCAGT
*ACTB*_ forward	TTGTTACAGGAAGTCCCTTGCC
*ACTB*_ reverse	ATGCTATCACCTCCCCTGTGTG
*MECOM*_forward	GACCCTTTGGCTAGATTGCTT
*MECOM*_reverse	CATGGGGATAGTCTTCGCTC
*CACNB2*_forward	TGCTCATGCCTCTTACCTGG
*CACNB2*_reverse	TCGGATGGACGGCTAGTGTA
*FLT1*_forward	CTGCTCAGCTGTCTGCTTCT
*FLT1*_reverse	GCCAGTGTGGTTTGCTTGAG
*IGF2*_forward	AAAAGTACAACATCTGGCCCGC
*IGF2*_reverse	AGAAGCACCAGCATCGACTT
*JUN*_forward	GCCAGGTCGGCAGTATAGTC
*JUN*_reverse	GGACTCTGCCACTTGTCTCC
*MKNK2*_forward	TCTCGGGCAGGTTTGAAGAC
*MKNK2*_reverse	CTCCACCTCCCTGAAAACCC
*DUSP8*_forward	GCTTCCTCTCCATCCTGCTG
*DUSP8*_reverse	GGCGTTGAGGACGTAGCTTA
*PDGFA*_forward	CAGCGACTCCTGGAGATAGAC
*PDGFA*_reverse	CAGATCAGGAAGTTGGCGGA

### Western blot analysis

Total protein was extracted from the cells using RIPA lysis buffer, separated via SDS-PAGE and subsequently transferred to PVDF membranes (Millipore, Billerica, USA). The membranes were then blotted with antibodies recognizing TXNIP (14715S, diluted 1:1000; CST, Beverly, USA), Erk1/2 (4695S, diluted 1:1000; CST), pErk1/2 (4370S, diluted 1:2000; CST), and β-actin (60008-1, diluted 1:5000; Proteintech, Chicago, USA). Protein bands were visualized and quantified with a Clinx Chemiluminescence System (Tanon, Shanghai, China).

### Pancreatic cancer mouse model

A lentivirus (CMV-TXNIP-EF1α-Puro) carrying human *TXNIP* was constructed and used to stably transfect PANC-1 and SW1990 cells. PANC-1 or SW1990 WT/TXNIP cells (2×10 ^6^) were subcutaneously implanted into BALB/c nude mice (GemPharmatech, Nanjing, China). Thirty days after cell inoculation, the mice were sacrificed and the tumors were removed, and tumor size and weight were measured. All experimental procedures were approved by the Institutional Animal Care and Use Committee of Fudan University Shanghai Cancer Center. Tumor tissues were subjected to immunohistochemical staining for Ki-67 and TUNEL staining. 

### TUNEL staining

To detect apoptosis, tissues were stained using the TUNEL assay kit (Servicebio, Wuhan, China). The methodology employed for TUNEL staining was described previously [17]. 

### Bioinformatics and statistical analysis

The PACA-AU dataset for PDAC was acquired from the International Cancer Genome Atlas (ICGC) database. A total of 245 patients with survival data in the database were enrolled in this study. The survminer R package was used to calculate the cut-off value for subsequent survival analysis. For the bulk RNA-seq data, differential expression analysis was performed utilizing the DESeq2 R package, and KEGG enrichment analysis of the DEGs was conducted using the ClusterProfiler R package.

Quantitative data are expressed as the mean±standard deviation (SD) and were analyzed based on variance and Student’s *t* tests. Chi-square tests were performed to compare TXNIP expression and clinical features. Univariate and multivariate analyses were also conducted on the basis of the log-rank test and the Cox proportional hazard model. *P* values less than 0.05 were considered to indicate statistical significance. 

## Results

### Patient characteristics

The clinicopathological characteristics of 218 patients with PDAC are presented in Table 3. The median age was 62 years (range 31‒84). Fifty-seven percent of patients were males, and most patients had lesions located on the head of the pancreas. Fifty-nine percent of patients had TNM stage II (103 patients) or III (26 patients) disease, and the median tumor diameter was 3.5 cm. None of the patients received neoadjuvant therapy. The median OS time was 15.8 months.

**Table:** **Table 3** Clinicopathologic characteristics of PDAC patients

Variables	*n*	TXNIP low	TXNIP high	*P* value
Total number	218	85 (39.0%)	133 (41.0%)	‒
Male gender	126	53 (42.1%)	73 (57.9%)	0.276
Female gender	92	32 (34.8%)	60 (65.2%)	
Age ≥ 65 years	71	27 (38.0%)	44 (62.0%)	0.840
Location				0.884
Head	127	49 (38.6%)	78 (61.4%)	
Body or tail	91	36 (39.6%)	55 (60.4%)	
Diameter (cm)				0.200
< 4	135	48 (35.6%)	87 (64.4%)	
≥ 4	83	37 (44.6%)	46 (55.4%)	
CA 19-9				0.667
< 37	48	20 (41.7%)	28 (58.3%)	
≥ 37	170	65 (38.2%)	105 (61.8%)	
TNM stage				0.535
I	89	31 (34.8%)	58 (65.2%)	
II	103	44 (42.7%)	59 (57.3%)	
III	26	10 (38.5%)	16 (61.5%)	
Grade				0.822
I	83	34 (41.0%)	49 (59.0%)	
II	117	45 (38.5%)	72 (61.5%)	
III	18	6 (33.3%)	12 (66.7%)	

### High expression of TXNIP is associated with improved outcomes in PDAC patients

**Figure 1:**
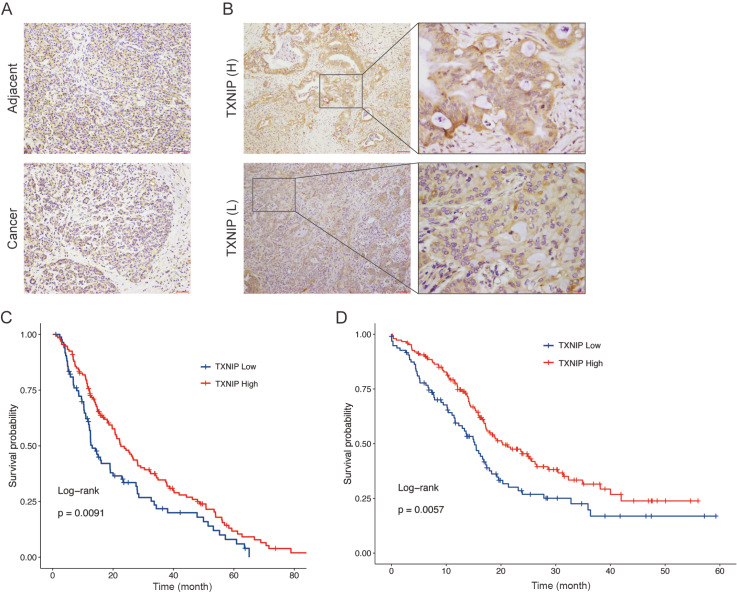
Figure 1 Immunostaining of TXNIP in PDAC tissues (A) Immunohistochemical evaluation of TXNIP expression in pancreatic cancer tissue and adjacent normal tissue. (B) The expression of TXNIP in the FUSCC cohort. (C) Increased TXNIP expression in tissue is associated with extended survival in PDAC patients. (D) High TXNIP level is correlated with improved prognosis in the ICGC-PACA-AU cohort.

To evaluate the clinical significance of TXNIP in pancreatic cancer, we examined the expression status of TXNIP by IHC staining. The expression of TXNIP was slightly greater in adjacent noncancerous tissue than that in tumor tissue ( Figure 1A). Then, the tumor tissue samples were divided into a high TXNIP group ( *n*=133) and a low TXNIP group ( *n*=85) based on the IHC intensities of TXNIP ( Figure 1B). Correlation analysis between TXNIP expression and clinicopathological characteristics demonstrated that TXNIP status was not associated with most clinicopathological variables, such as age, sex, tumor location, tumor diameter, CA 19-9 concentration, TNM stage or histological grade ( Table 3). Moreover, univariate analysis revealed that the variables associated with OS were tumor diameter [hazard ratio (HR)=1.551, *P*=0.005] and TNM stage (HR=1.634, 1.738; *P*=0.003, 0.034). Patients with high TXNIP expression had prolonged OS (HR=0.667, *P*=0.009; Table 4 and Figure 1C). We also performed multivariate analysis to determine whether TXNIP expression is still an independent prognostic indicator of OS. TXNIP expression, tumor diameter and TNM stage were subjected to multivariate analysis. We found that tumor diameter (HR=1.471, *P*=0.014) and TXNIP expression (HR=0.708, *P*=0.029) were independent predictors of OS. In addition, we validated our findings using the ICGC-PACA-AU database, which contains sequencing data and prognostic information for 245 patients. The optimal cut-off value for TXNIP expression was calculated to be 12.83 using the survminer R package, and patients were stratified into high expression ( *n*=149) and low expression ( *n*=96) groups based on the cut-off value. Log-rank survival analysis revealed that patients with high TXNIP expression had a significantly better prognosis than those with low TXNIP expression ( Figure 1D).

**Table:** **Table 4** Survival analysis for PDAC patients

Variables	*n*	Univariate analysis		Multivariable analysis
HR	95% CI	*P* value		HR	95% CI	*P* value
Gender								
Female	126	1						
Male	92	1.102	0.816–1.489	0.526				
Age years								
< 65	147	1						
≥ 65	71	1.326	0.960–1.832	0.087				
Location								
Head	127	1						
Body or tail	91	0.939	0.696–1.268	0.682				
Diameter (cm)								
< 4	135	1				1		
≥ 4	83	1.551	1.144–2.103	**0.005**		1.471	1.081‒2.001	**0.014**
CA19-9								
< 37	48	1						
≥ 37	170	1.020	0.711–1.462	0.915				
TXNIP								
Low	85	1				1		
High	133	0.667	0.491–0.906	**0.009**		0.708	0.519‒0.966	**0.029**
TNM stage								
I	89	1				‒	‒	‒
II	103	1.634	1.187–2.249	**0.003**		‒	‒	‒
III	26	1.738	1.043–2.895	**0.034**		‒	‒	‒
Grade								
I	83	1						
II	117	0.777	0.568–1.063	0.114				
III	18	0.626	0.357–1.097	0.102				

HR, hazard ratio; CI, confidence interval. Significant difference is shown in boldface.

### Increased TXNIP expression suppresses the malignant potential of pancreatic cells

**Figure 2:**
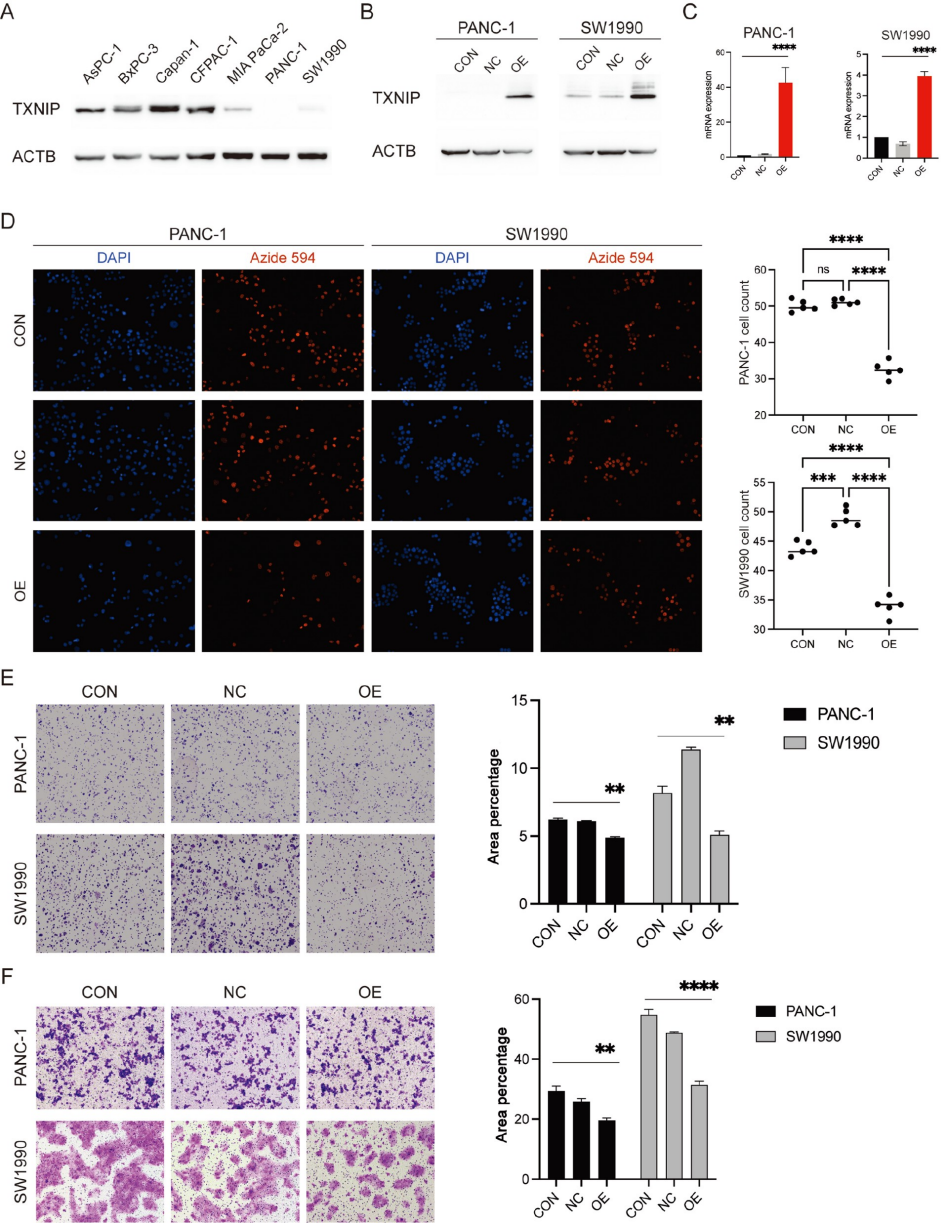
Figure 2 Increased TXNIP expression suppresses the malignancy of pancreatic cancer cells (A) Western blot analysis of TXNIP expression in PDAC cell lines. TXNIP was upregulated in PANC-1 and SW1990 cells, as evaluated by (B) western blot analysis and (C) RT-qPCR analysis. (D) Cell proliferation was detected by EdU incorporation assay in PANC-1 and SW1990 cells with or without TXNIP overexpression (blue: nuclei; red: nuclei with incorporated EdU). (E) Clonogenic assays in PANC-1 and SW1990 cells demonstrating reduced colony formation following TXNIP overexpression. (F) Cell migration assays of PANC-1 and SW1990 cells showing decreased cell motility after TXNIP overexpression. Data are presented as the mean±SD of five experiments. Two-way ANOVA was used. **P<0.01, ***P<0.001, ****P<0.0001, ns, not significant.

To elucidate the role of the tumor suppressor gene *TXNIP* in the pathogenesis of pancreatic cancer, we initially quantified its expression in a panel of pancreatic cancer cell lines. We observed high expression of TXNIP in the AsPC-1, BxPC-3, Capan-1, and CFPAC-1 cell lines, in contrast to the relatively low expression in the MIA PaCa-2, PANC-1, and SW1990 cell lines ( Figure 2A). Hence, we opted to overexpress TXNIP in the PANC-1 and SW1990 cell lines ( Figure 2B,C). To elucidate the impact of TXNIP on tumorigenesis, we performed EdU cell proliferation and colony formation assays to evaluate DNA synthesis and colony formation capabilities. Notably, EdU proliferation and colony formation assays demonstrated that overexpression of TXNIP inhibited the proliferation of PANC-1 and SW1990 cells ( Figure 2D,E). Subsequently, we employed Transwell migration assays to probe the potential influence of TXNIP on the migratory behavior of pancreatic cells. Overexpression of TXNIP suppressed the migration of PANC-1 and SW1990 cells ( Figure 2F). However, overexpression of TXNIP did not noticeably influence the apoptosis or cell cycle progression of PANC-1 or SW1990 cells ( Supplementary Figure S1A,B). Moreover, the difference in the sensitivity of these two cell lines to gemcitabine was not significant ( Supplementary Figure S1C). No significant differences were observed in the C11 staining of the two tumor cell lines, whether the ferroptosis inducers RSL3 (1 μM) or erastin (5 μM) were used ( Supplementary Figure S1D). Collectively, these findings indicated that *in vitro* overexpression of TXNIP could attenuate tumorigenic processes in pancreatic cancer cells.

### TXNIP inhibits tumor proliferation *in vivo*

**Figure 3:**
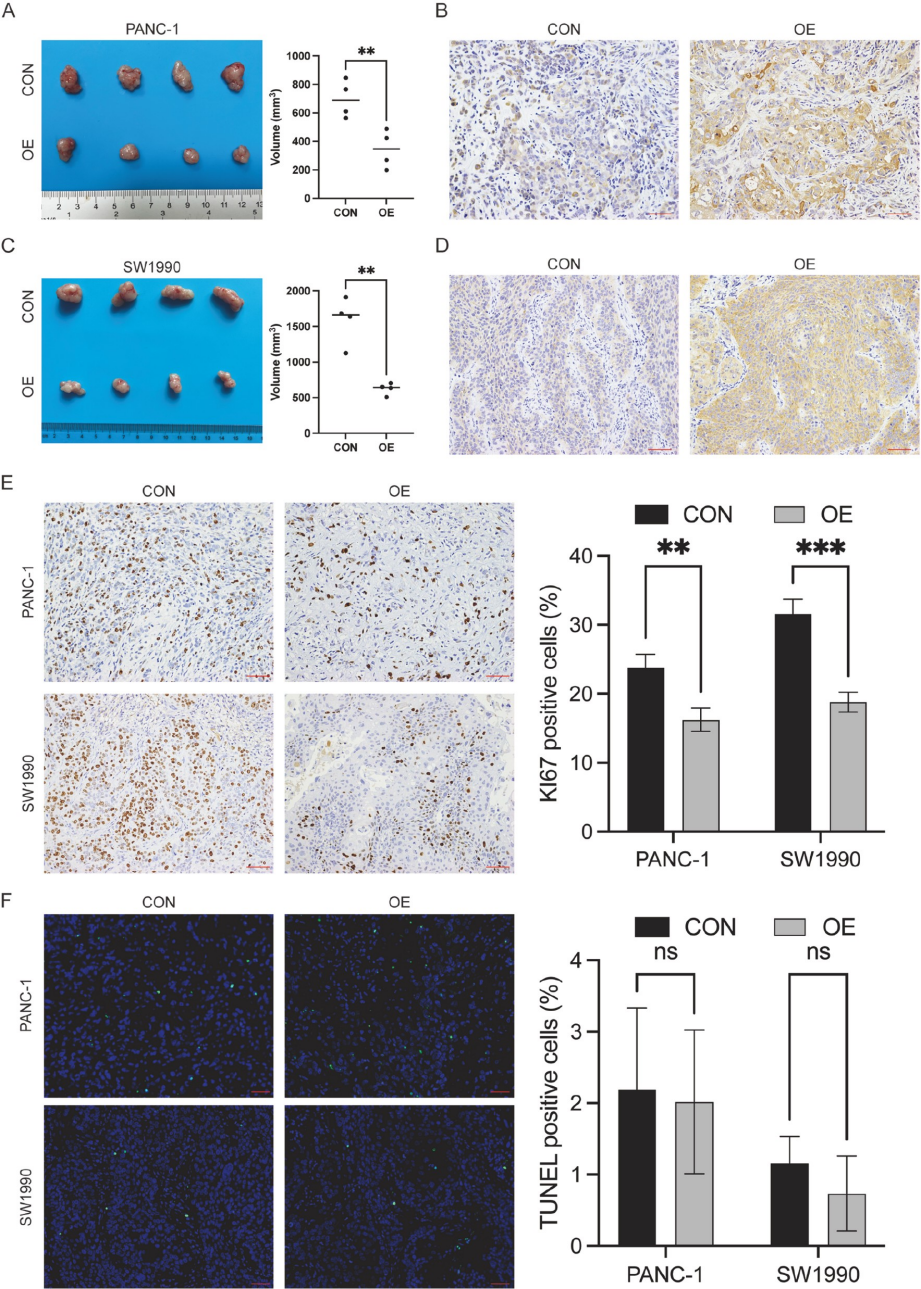
Figure 3 TXNIP overexpression inhibits tumor proliferation *in vivo* (A,C) Representative images of nude mouse xenografts derived from PANC-1 and SW1990 cells. Each group contained four animals. Tumor volume was measured and compared using Student’s t test. (B,D) Illustrative examples of TXNIP staining of PANC-1 or SW1990 tumors from nude mice. (E,F) Xenograft tumor tissues were stained for Ki-67 and subject to TUNEL staining. Scale bar: 50 μm (red line at the bottom right). Ki-67-positive (brown) or TUNEL-positive cells (green) were quantified and statistically analyzed by Student’s t test. Data are presented as the mean±SD. **P<0.01, ***P<0.001, ns, not significant.

To determine the *in vivo* influence of TXNIP on tumorigenesis, PANC-1 and SW1990 cells with or without TXNIP overexpression were subcutaneously injected into nude mice. We subsequently assessed the proliferation potential of the cells *in vivo*. We observed that the subcutaneous tumor volume in nude mice was noticeably smaller following overexpression of the TXNIP gene than in the control group ( Figure 3A,C). IHC results also confirmed increased TXNIP expression in the subcutaneous tumors of the overexpression group ( Figure 3B,D). Furthermore, we found a greater proportion of proliferating tumor cells in the control group ( Figure 3E). However, there were no significant differences in the proportion of apoptotic cells in the tumor tissues ( Figure 3F). These findings suggest that TXNIP may suppress the malignant potential of tumors by inhibiting tumor cell proliferation.

### TXNIP inhibits tumor proliferation via the MAPK signaling pathway

**Figure 4:**
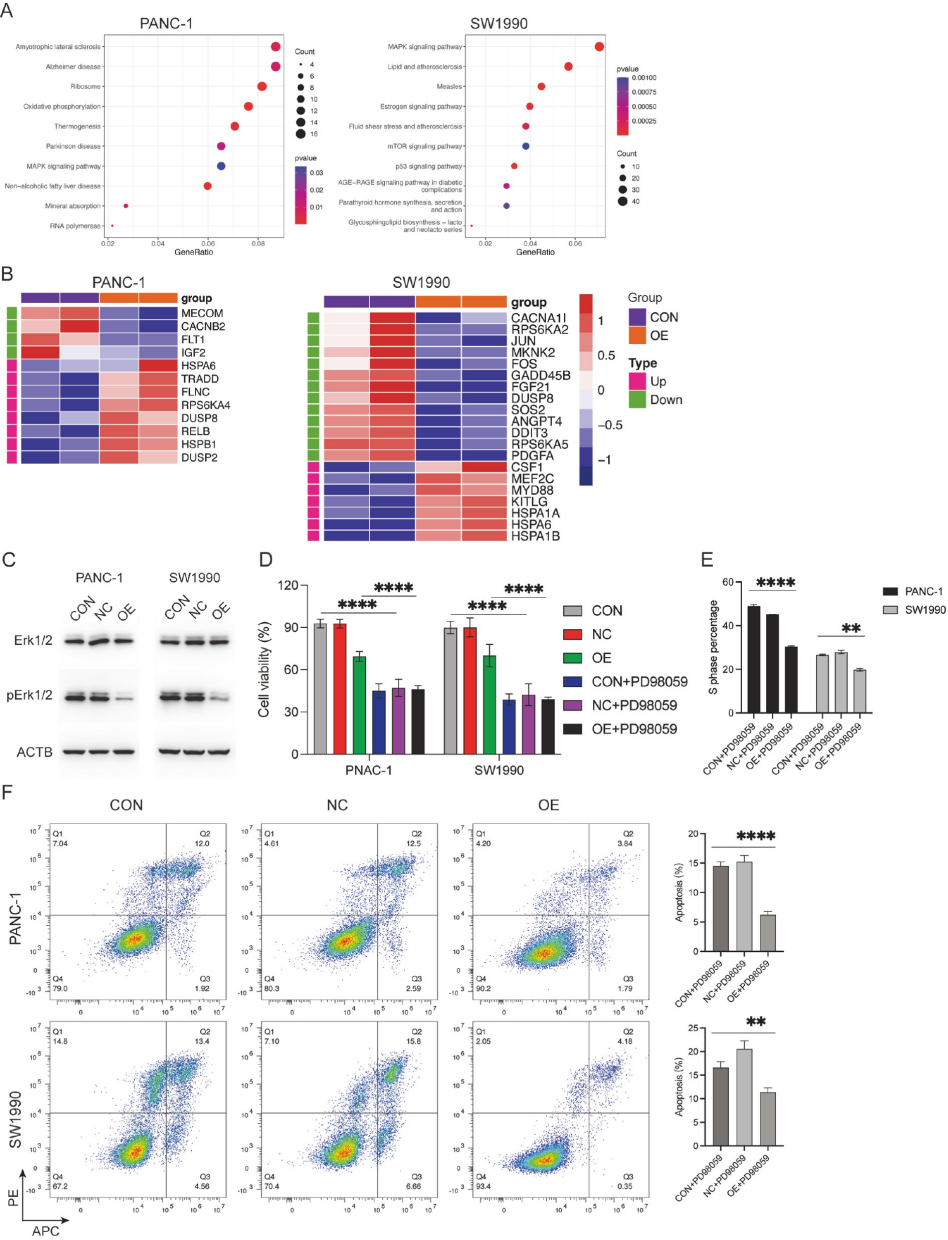
Figure 4 TXNIP-related functions are mediated by the MAPK signaling pathway (A) KEGG enrichment analysis revealed enrichment of the MAPK signaling pathway in both cell groups. (B) Heatmap showing the genes associated with the MAPK signaling pathway. (C) Western blot analysis of Erk1/2 and pErk1/2 expressions in PANC-1 and SW1990 cells following TXNIP overexpression. (D) TXNIP-overexpressing PANC-1 and SW1990 cells were treated with PD98059 (20 μM) for 24 h, after which cell viability was determined. (E,F) The cell cycle and apoptosis were analyzed in TXNIP-overexpressing PANC-1 and SW1990 cells after treatment with PD98059 (20 μM) for 48 h. **P<0.01, ****P<0.0001.

To further elucidate the specific mechanisms by which TXNIP influences tumor cell proliferation, we employed RNA sequencing (RNA-seq) to analyze the impact of TXNIP overexpression on the gene expression profiles of PANC-1 and SW1990 cells. Following differential analysis of the sequencing data, we found that TXNIP was significantly overexpressed in both cell groups ( Supplementary Figure S2A), consistent with our preliminary validation data. Upon performing KEGG enrichment analysis of the DEGs using the clusterProfiler R package, we found that both cell groups were enriched in the MAPK signaling pathway ( Figure 4A). However, the differentially expressed genes in this pathway did not show consistent up- or downregulation across the two cell groups ( Figure 4B). Verification of the significant DEGs in both cell groups yielded results consistent with the sequencing data (Supplementary Figure S2B). Thus, it is apparent that the *TXNIP* gene does not regulate this pathway by influencing any specific gene within the MAPK signaling pathway. To further clarify the impact of TXNIP on the MAPK signaling pathway, we used western blot analysis to validate changes in the phosphorylation levels of key proteins within this pathway. We found that the phosphorylation level of the Erk1/2 protein was significantly reduced in the overexpression group, while the total level of the Erk1/2 protein was not significantly decreased ( Figure 4C). Moreover, after treatment with the Erk inhibitor (20 μM), the proliferative ability of the control cells was significantly inhibited, while no significant differences were observed in the overexpression group compared with the control group ( Figure 4D). In addition, the Erk inhibitor caused S-phase arrest and cell apoptosis in PANC-1 and SW1990 cells, with a more pronounced effect on the control cells. ( Figure 4E,F). Therefore, we propose that TXNIP inhibits the activation of the MAPK signaling pathway by suppressing the phosphorylation of the Erk1/2 protein in tumor cells, thereby inhibiting tumor cell proliferation.

### Silencing of *TXNIP* affects the invasiveness of pancreatic cancer cells 

**Figure 5:**
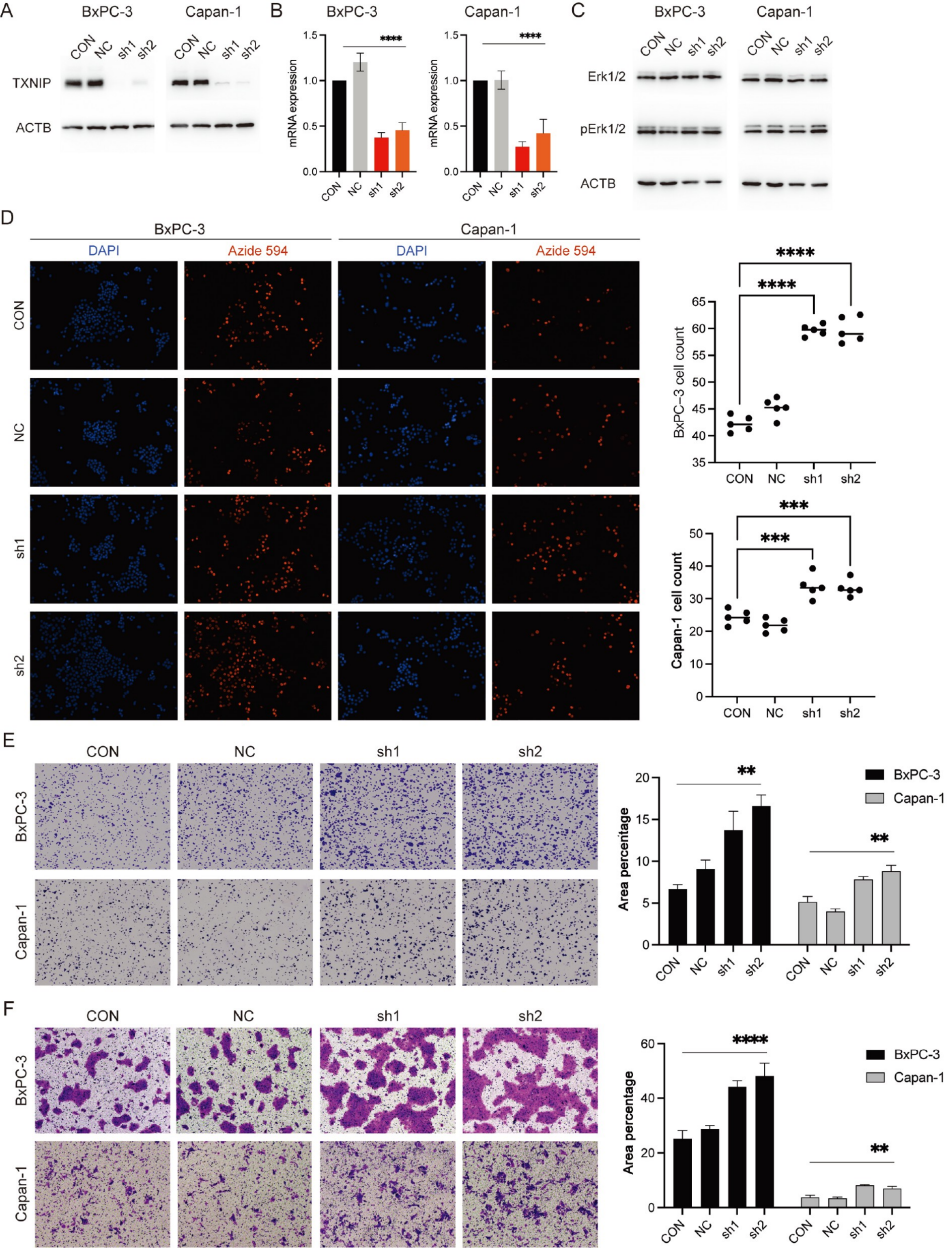
Figure 5 Inhibition of TXNIP enhances the invasion of pancreatic cancer cells TXNIP was silenced in BxPC-3 and Capan-1 cells, after which TXNIP expression was evaluated via (A) western blot analysis and (B) RT-qPCR. (C) Western blot analysis of Erk1/2 and pErk1/2 expressions in TXNIP-silenced BxPC-3 and Capan-1 cells. (D) Proliferation of BxPC-3 and Capan-1 cells following TXNIP silencing, as determined by EdU incorporation assay. (E) Clonogenic assays in BxPC-3 and Capan-1 cells demonstrating enhanced colony formation following TXNIP silencing. (F) Cell migration assays of BxPC-3 and Capan-1 cells showing increased cell motility after TXNIP silencing. **P<0.01, ***P<0.001, ****P<0.0001.

**Figure 6:**
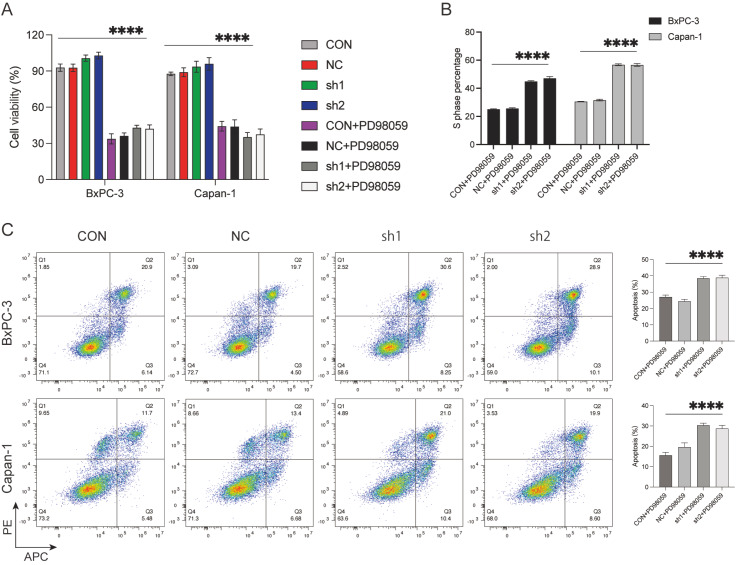
Figure 6 Effect of the Erk inhibitor on *TXNIP*-silenced pancreatic cells (A) TXNIP-silenced BxPC-3 and Capan cells were treated with PD98059 (20 μM) for 24 h. Then, cell viability was determined by CCK-8 assay. (B,C) The cell cycle and apoptosis were assessed in TXNIP-silenced BxPC-3 and Capan-1 cells following treatment with PD98059 (20 μM) for 48 h. ****P<0.0001.

Furthermore, we examined the effect of *TXNIP* knockdown on the malignant potential of tumor cells. *TXNIP* was silenced in BxPC-3 and Capan-1 cells ( Figure 5A,B), and both EdU proliferation and colony formation assays indicated notable enhancement of tumor cell proliferation following *TXNIP* knockdown ( Figure 5D,E). Transwell cell migration assays suggested that *TXNIP* knockdown significantly increased the migratory ability of tumor cells ( Figure 5F). However, the cell cycle and apoptosis assays did not show any significant differences in BxPC-3 and Capan-1 cells after silencing of *TXNIP* ( Supplementary Figure S3A,B). We also observed an increase in the phosphorylation level of the Erk1/2 protein in tumor cells after *TXNIP* knockdown ( Figure 5C). Erk inhibitor treatment significantly suppressed the proliferative activity of these cells with *TXNIP* knockdown ( Figure 6A). In addition, the Erk inhibitor caused S phase arrest and cell apoptosis in BxPC-3 and Capan-1 cells, with a more significant effect on the *TXNIP*-silenced cells ( Figure 6B,C).

## Discussion

TXNIP has diverse biological functions in humans, such as modulating endocrine metabolism, regulating intracellular gene transcription, controlling numerous stress responses, and contributing to natural killer cell development [ 9, 19]. Previous research [ 20, 21] has demonstrated that TXNIP expression is downregulated in a range of tumor tissues, and aberrant TXNIP expression is linked to a favorable patient prognosis. Our own investigation further corroborated these findings, as we observed improved outcomes in patients with elevated TXNIP expression. 

We subsequently investigated the influence of TXNIP on pancreatic cancer cells. Our findings revealed that TXNIP overexpression in PANC-1 and SW1990 cells markedly diminished their proliferative and migratory capabilities, which is consistent with a previous study in osteosarcoma [22]. Similarly, our *in vivo* experiments confirmed that TXNIP overexpression suppresses tumor cell proliferation and restricts tumor growth. However, TXNIP inhibition of thioredoxin potentially decreases the capacity of cells to eliminate ROS, potentially inducing intracellular oxidative damage [ 23, 24]. Hang *et al*. [25] revealed that TXNIP-driven oxidative damage could induce ferroptosis in renal tubular epithelial cells, exacerbating renal injury. Moreover, Jian *et al*. [15] found that TXNIP-triggered oxidative damage could induce apoptosis in liver cancer cells. However, in the context of pancreatic cancer, we detected no accumulation of lipid peroxides or any difference in apoptosis levels in cells overexpressing TXNIP compared to those in control cells. Thus, in pancreatic cancer, the tumor-suppressive effects of TXNIP do not stem from its inhibition of ROS clearance. 

To further delineate the potential mechanisms through which TXNIP inhibits cancer progression in pancreatic cancer, we utilized transcriptome sequencing to analyze the differential gene expression profiles of tumor cells before and after TXNIP overexpression. Our findings revealed enrichment of differentially expressed genes in the MAPK signaling pathway. Previous studies indicated that the MAPK signaling pathway is involved in various cellular functions, such as proliferation, differentiation, and migration [ 26, 27]. In pancreatic cancer, inhibiting the activity of the MAPK signaling pathway can suppress the malignant potential of tumor cells, including their ability to proliferate and migrate [ 28, 29]. Within the MAPK signaling pathway, the Erk protein branch is closely related to cell proliferation and differentiation [30]. Inhibiting Erk protein phosphorylation can suppress the proliferative ability of tumor cells. We further explored the Erk protein family and found that after TXNIP overexpression, the phosphorylation level of the Erk1/2 protein in cells was significantly reduced without affecting the total protein expression level of Erk1/2. Therefore, we infer that TXNIP can inhibit the activity of the MAPK signaling pathway and suppress the proliferative ability of tumor cells by inhibiting the phosphorylation of the Erk1/2 protein. 

In summary, our findings establish TXNIP as a potential prognostic indicator and treatment target for pancreatic cancer. The physiological roles of TXNIP in pancreatic cancer warrant further investigation with the aim of enhancing the control of invasion and metastasis and ultimately improving the prognosis for this destructive disease.

## Supporting information

23384supplementary_figures

## Supplementary Data

Supplementary data is available at *Acta Biochimica et Biophysica Sinica* online.
