# Supplementary material for: Suppression of pancreatic cancer proliferation through TXNIP-mediated inhibition of the MAPK signaling pathway: TXNIP inhibits pancreatic cancer via the MAPK pathway
Source: Acta Biochim Biophys Sin (Shanghai). 2024 Jan 16;56(4):513–24. doi: 10.3724/abbs.2023286 (PMC11094629; doi:10.3724/abbs.2023286)
Supplement: 23384supplementary_figures [file 23384supplementary_figures.pdf]

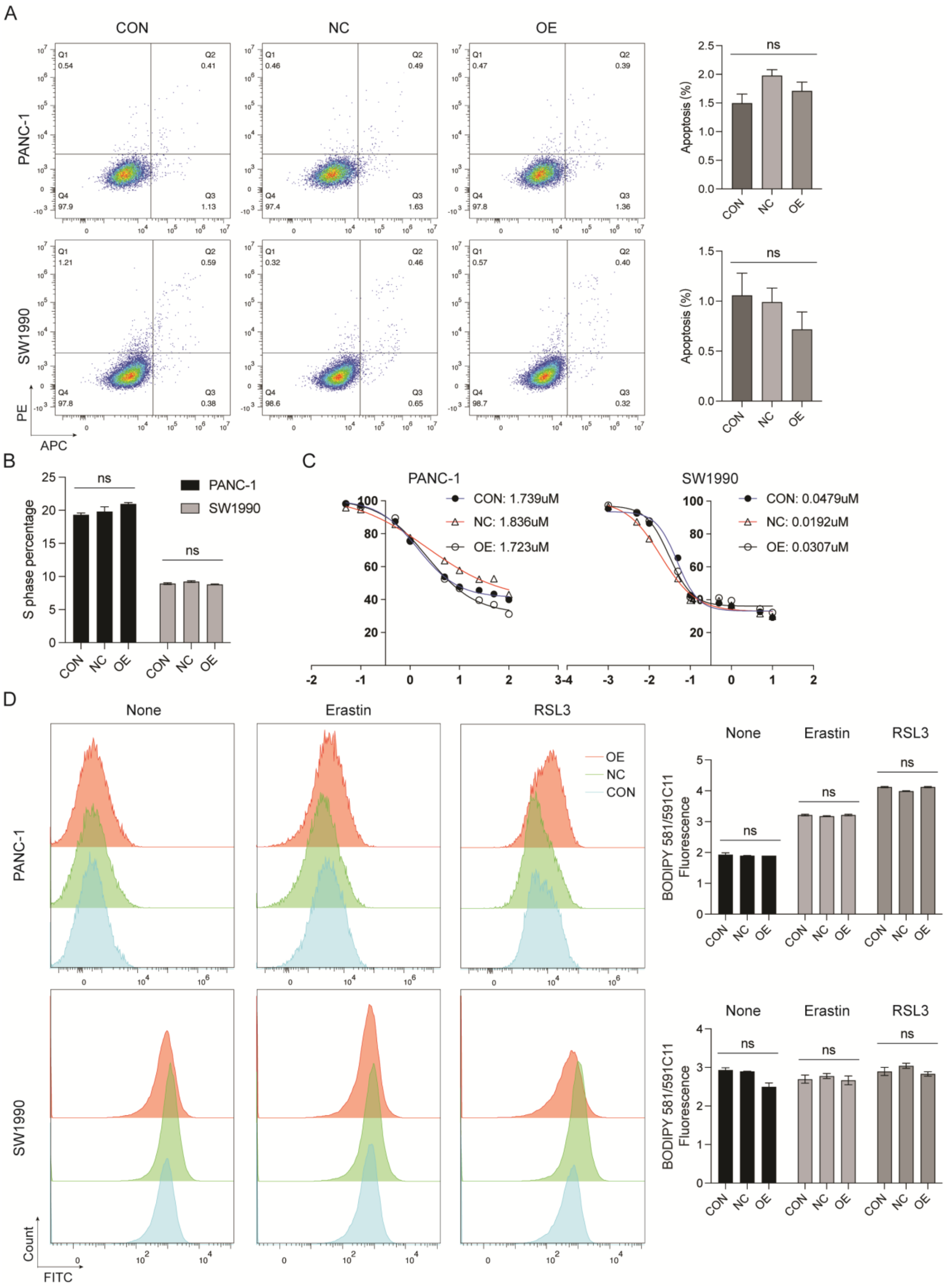
A
CON
NC
OE
PANC-1
SW1990
PE
APC
Q1 0.54
Q2 0.41
Q4 97.9
Q3 1.13
Q1 0.46
Q2 0.49
Q4 97.4
Q3 1.63
Q1 0.47
Q2 0.39
Q4 97.8
Q3 1.36
Q1 1.21
Q2 0.59
Q4 97.8
Q3 0.38
Q1 0.32
Q2 0.46
Q4 98.6
Q3 0.65
Q1 0.57
Q2 0.40
Q4 98.7
Q3 0.32
Apoptosis (%)
ns
B
S phase percentage
PANC-1
SW1990
ns
C
PANC-1
CON: 1.739uM
NC: 1.836uM
OE: 1.723uM
SW1990
CON: 0.0479uM
NC: 0.0192uM
OE: 0.0307uM
D
None
Erastin
RSL3
OE
NC
CON
Count
FITC
BODIPY 581/591C11 Fluorescence
ns

Supplementary Figure S1. Overexpression of TXNIP does not significantly influence apoptosis, the cell cycle, ferroptosis, or sensitivity to gemcitabine in PANC-1 or SW1990 cells (A) Assessment of apoptosis rates in PANC-1 and SW1990 cells. (B) Cell cycle analysis of PANC-1 and SW1990 cells. (C) The IC_{50} for cell sensitivity to the chemotherapeutic agent gemcitabine did not substantially differ between control and TXNIP-overexpressing cells. (D) C11 staining of cells from both cell lines following treatment with the ferroptosis inducers RSL3 (1 μM) or erastin (5 μM) showed no significant differences.

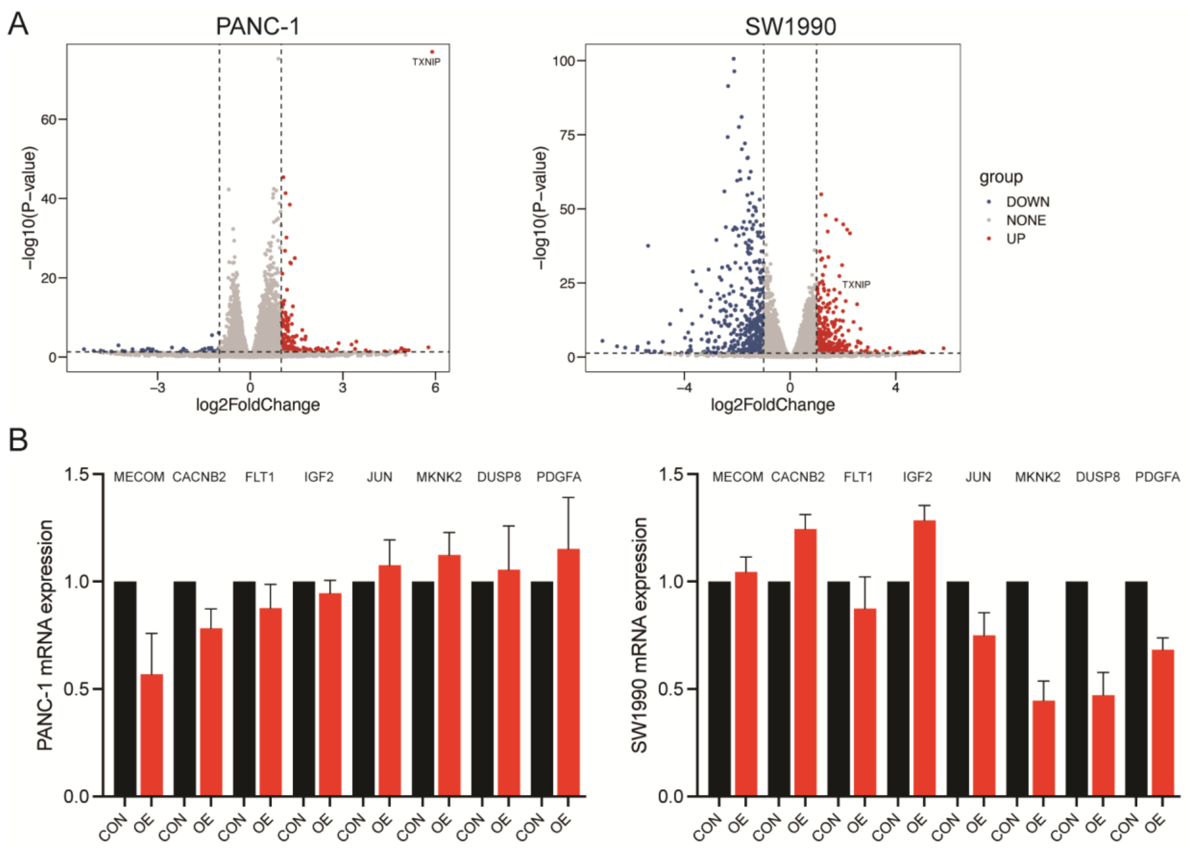

Supplementary Figure S2. TXNIP was significantly overexpressed and significant DEGs was confirmed (A) Volcano plot showing significant overexpression of TXNIP in PANC-1 and SW1990 cells. (B) RT-qPCR was performed to evaluate the genes associated with the MAPK signaling pathway.

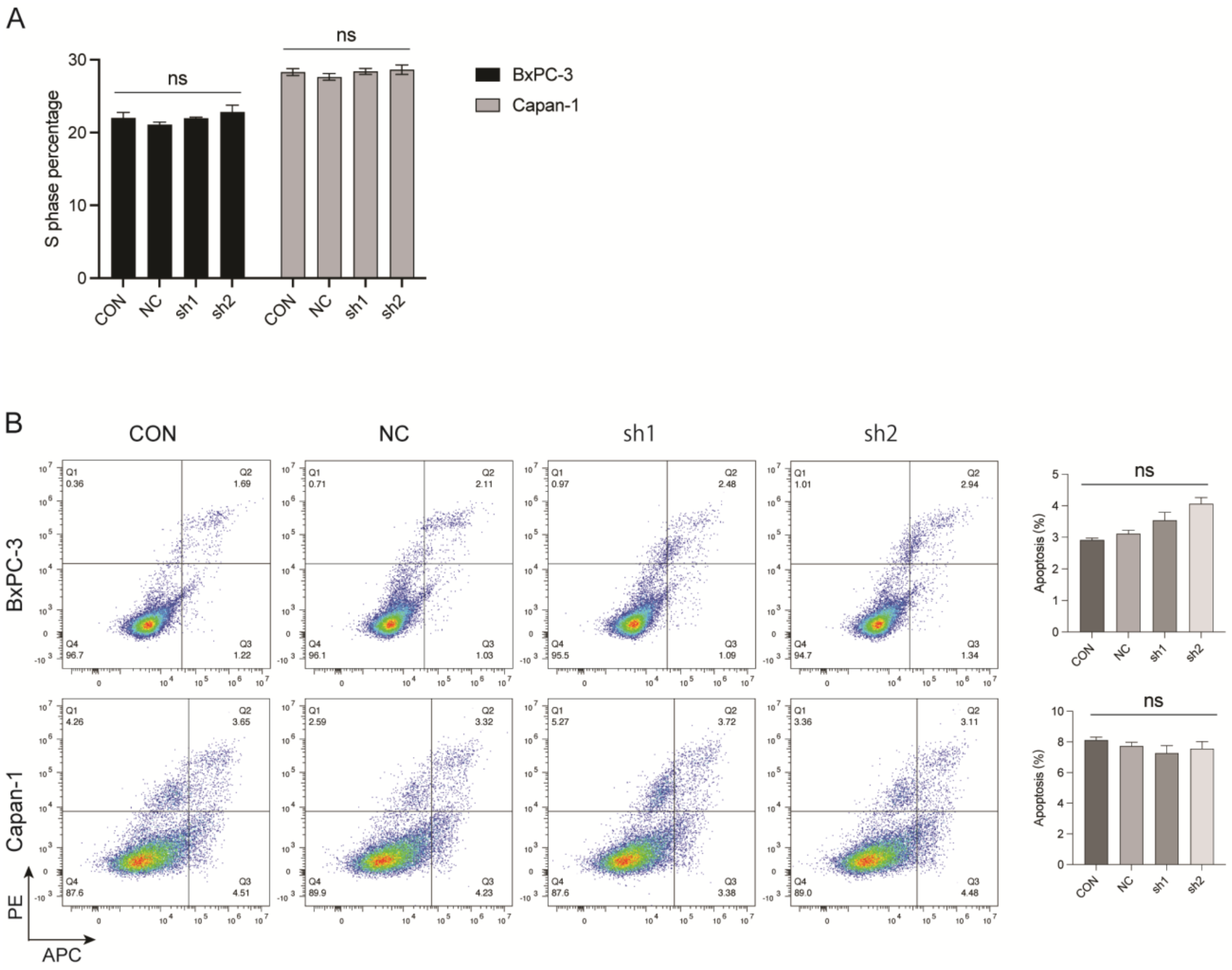

Supplementary Figure S3. **Inhibition of TXNIP does not significantly influence apoptosis or cell cycle in BxPC-3 or Capan-1 cells** (A,B) The cell cycle and apoptosis were assessed in *TXNIP*-silenced BxPC-3 and Capan cells.
